# Myocardial ischemia induced by major aortopulmonary collateral arteries treated with transcatheter coil embolization

**DOI:** 10.1002/ccr3.980

**Published:** 2017-05-12

**Authors:** Ryota Kakizaki, Taiki Tojo, Yoshiyasu Minami, Toshimi Koitabashi, Reiko Woodhams, Takao Shimohama, Masahiro Ishii, Junya Ako

**Affiliations:** ^1^ Department of Cardiovascular MedicineKitasato University School of MedicineSagamiharaJapan; ^2^ Department of Diagnostic RadiologyKitasato University School of MedicineSagamiharaJapan; ^3^ Department of PediatricsKitasato University School of MedicineSagamiharaJapan

**Keywords:** Coil embolization, coronary steal, major aortopulmonary collateral arteries

## Abstract

Major aortopulmonary collateral arteries branching from coronary arteries may cause coronary steal. The careful follow‐up is needed irrespective of symptoms because increasing physical activities and oxygen demand along with the age may induce myocardial ischemia. Transcatheter intervention by well‐trained physician would be a treatment option in patients with myocardial ischemia.

## Introduction

Major aortopulmonary collateral arteries (MAPCAs) are systemic‐to‐pulmonary fistulae branching from aorta or other arteries to pulmonary arteries. MAPCAs are remnants of the embryonic ventral splanchnic arteries which concomitantly regress with the formation of the normal pulmonary arterial system in the first week of gestation. In patients with tetralogy of Fallot (TOF) and pulmonary atresia, the incidence of MAPCAs is reported as 1% 1, and fistulae provide an alternative pulmonary blood supply 2. On the other hand, coronary artery fistula may cause myocardial ischemia due to coronary steal though the number of previous reports is limited 3. Therefore, the clinical manifestation and optimal treatment strategy remain unknown.

## Case Report

**Figure 1:**
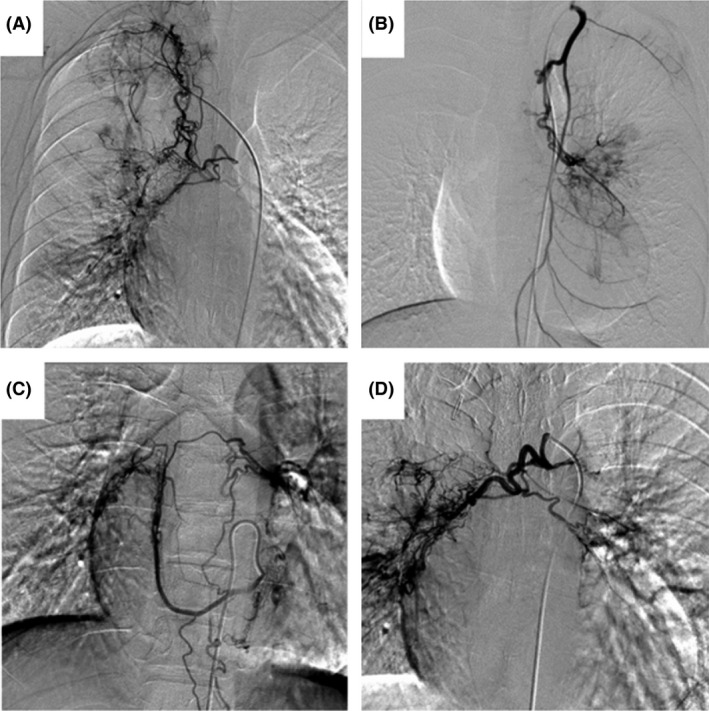
Systemic‐to‐pulmonary fistulae branching from aorta and other major arteries. Fistulae from right (A) and left (B) internal mammary artery, left gastroepiploic artery, left bronchial artery, left intercostal artery (C), and aortic arch (D) to pulmonary arteries were observed on angiogram.

**Figure 2:**
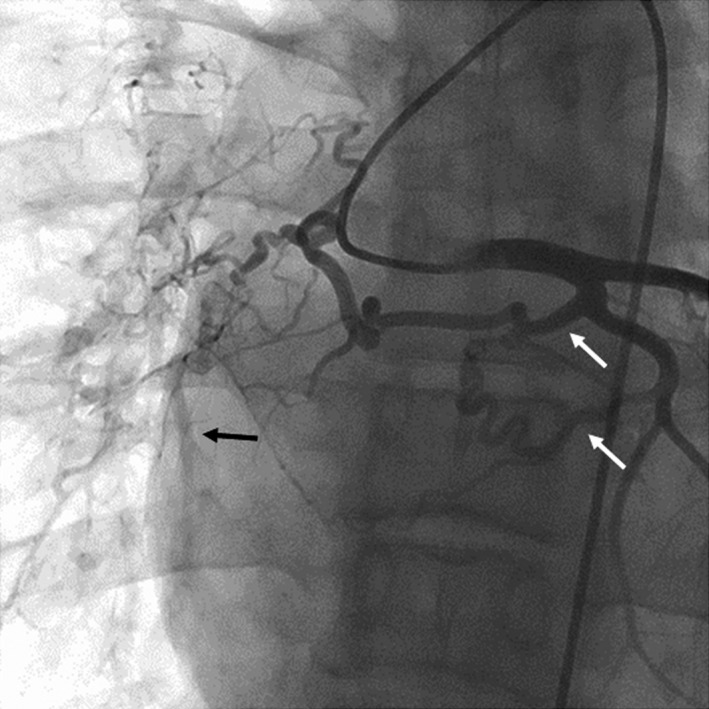
Coronary artery fistulae barnching from left circumflex artery. Coronary angiography showed two major fistulae (white arrows) from left circumflex artery (LCx) to pulmonary arteries. Right pulmonary artery (black arrow) was imaged via fistula branching proximal of LCx.

**Figure 3:**
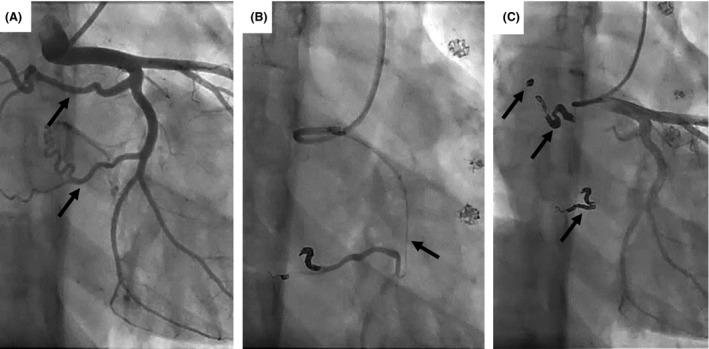
Transcatheter coil embolization of coronary artery fistulae. Two major coronary artery fistulae (arrow) from left circumflex to left and right pulmonary artery were imaged (A). Coils were inserted into fistulae via micro catheter (arrow) (B). After embolization using a total of seventeen coils (arrow), the fistulae were disappeared (C).

An 18‐year‐old male was admitted due to recurrent anterior chest pain and dyspnea on exercise. He had surgical history of right Blalock‐Taussig shunt at 1 year old and cardiac repair for TOF at 2 years old. Although he had been diagnosed as MAPCAs, surgical intervention termed “unifocalization” had not been performed because sufficient pulmonary artery flow had been observed. Physical examination showed no significant signs of heart failure and cardiac murmur. Electrocardiography and blood test including cardiac enzymes showed no abnormal findings. Echocardiography showed no left ventricular dysfunction but moderate pulmonary valve regurgitation and mild tricuspid valve regurgitation without pulmonary hypertension. However, myocardial exercise stress perfusion scintigraphy demonstrated myocardial ischemia in left ventricular anterior wall. Because atherosclerotic coronary artery stenosis is rare in young patients, we suspected the presence of myocardial ischemia due to the structural abnormalities of coronary artery from his past medical history. On angiogram, systemic‐to‐pulmonary fistulae were still observed from multiple sites to pulmonary arteries (Fig. 1 A–D). Coronary angiography also showed two major coronary artery fistulae from left circumflex artery (LCx) to left and right pulmonary arteries (Fig. 2) without significant coronary artery stenosis. The diameter was 3.0 mm and 1.6 mm, respectively. Because coronary steal through fistulae was suspected as the cause of myocardial ischemia, we decided to perform transcatheter coil embolization of coronary artery fistulae. Through a 2.6 Fr microcatheter (FINECROSS MG^®^, TERUMO co., Tokyo, JAPAN) placed at coronary artery fistulae via LCx, a total of eleven 0.012‐inch coils (two 2.0 × 20 mm, five 2.5 × 50 mm, four 3 × 80 mm, Orbit Galaxy Detachable Coil System^®^, DePuy Synthes, West Chester, PA) and six 0.010‐inch coils (four 3.0 × 60 mm, two 3.0 × 40 mm, Target^®^, Stryker Neurovascular, Fremont, California) were implanted in coronary artery fistulae (Fig. 3B). The final coronary angiogram showed no abnormal blood flow from LCx (Fig. 3C). After the procedure, his chest symptoms completely disappeared.

## Discussion

Herein, we report a rare case of myocardial ischemia induced by coronary steal through fistulae in a young adult patient with MAPCAs, which were treated by transcatheter coil embolization. Although the optimal management of MAPCAs remains undetermined, transcatheter coil embolization successfully resolved myocardial ischemia in the present case.

One of major clinical concerns regarding coronary artery fistula including MAPCAs is myocardial ischemia due to coronary steal 3 which might cause acute myocardial infarction 4. Although coronary artery fistula could be spontaneously resolved 5, the majority of patients consulting a medical institution have some symptoms 6. Therefore, physicians might have to consider the surgical or transcatheter closure of persistent fistula. However, the management of coronary artery fistula has not been clearly standardized. There is no significant difference in the success rate and the incidence of complications between surgical approach and transcatheter approach 7, 8 though surgical approach requires longer hospitalization. Thus, the strategy of coronary artery fistula closure should be decided according to the individual anatomy of fistula and comorbidities though percutaneous approach might be better in case with the history of cardiac surgery.

In the present case, the patient had no ischemic driven symptom in his childhood. However, he suffered from chest pain in his adolescence, and myocardial ischemia was demonstrated in exercise stress perfusion scintigraphy. We speculated that the increased physical activities and oxygen demand along with his growth manifested the existence of coronary steal syndrome via persistent fistulae. Thus, physicians might have to consider annual noninvasive examination irrespective of symptom and immediate further examinations in symptomatic patients. In patients with MAPCAs, the careful follow‐up through life and surgical or transcatheter treatment should be considered if the patients suffer from myocardial ischemia.

## Conflict of Interest

None.

## Disclosures

None.

## Authorship

RK: Manuscript writing, TT: Conception of the work, YM: Manuscript writing, TK: Critical revision of the manuscript, RW: Data interruption, TS: Critical revision of the manuscript, MI: Critical revision of the manuscript, JA: Corresponding author, final approval of manuscript.

## Supporting information


**Video S1.** Coronary artery fistulae barnching from left circumflex artery. Angiography of left coronary artery showed two fistulae from left circumflex artery (LCx) to pulmonary arteries. Right pulmonary artery was imaged via fistula branching proximal of LCx.
